# Evaluation of reporting timeliness of public health surveillance systems for infectious diseases

**DOI:** 10.1186/1471-2458-4-29

**Published:** 2004-07-26

**Authors:** Ruth Ann Jajosky, Samuel L Groseclose

**Affiliations:** 1 Centers for Disease Control and Prevention, Epidemiology Program Office, Division of Public Health Surveillance and Informatics, Surveillance Systems Branch, Atlanta, Georgia, 30333, USA; 2 Centers for Disease Control and Prevention, National Center for HIV, STD, & TB Prevention, Division of STD Prevention, Statistics and Data Management Branch, Atlanta, Georgia, 30333, USA; 3 At the time this study was conducted this co-author was Chief of the Surveillance Systems Branch

## Abstract

**Background:**

Timeliness is a key performance measure of public health surveillance systems. Timeliness can vary by disease, intended use of the data, and public health system level. Studies were reviewed to describe methods used to evaluate timeliness and the reporting timeliness of National Notifiable Diseases Surveillance System (NNDSS) data was evaluated to determine if this system could support timely notification and state response to multistate outbreaks.

**Methods:**

Published papers that quantitatively measured timeliness of infectious disease surveillance systems operating in the U.S. were reviewed. Median reporting timeliness lags were computed for selected nationally notifiable infectious diseases based on a state-assigned week number and various date types. The percentage of cases reported within the estimated incubation periods for each disease was also computed.

**Results:**

Few studies have published quantitative measures of reporting timeliness; these studies do not evaluate timeliness in a standard manner. When timeliness of NNDSS data was evaluated, the median national reporting delay, based on date of disease onset, ranged from 12 days for meningococcal disease to 40 days for pertussis. Diseases with the longer incubation periods tended to have a higher percentage of cases reported within its incubation period. For acute hepatitis A virus infection, which had the longest incubation period of the diseases studied, more than 60% of cases were reported within one incubation period for each date type reported. For cryptosporidiosis, *Escherichia coli *O157:H7 infection, meningococcal disease, salmonellosis, and shigellosis, less than 40% of cases were reported within one incubation period for each reported date type.

**Conclusion:**

Published evaluations of infectious disease surveillance reporting timeliness are few in number and are not comparable. A more standardized approach for evaluating and describing surveillance system timeliness should be considered; a recommended methodology is presented. Our analysis of NNDSS reporting timeliness indicated that among the conditions evaluated (except for acute hepatitis A infection), the long reporting lag and the variability across states limits the usefulness of NNDSS data and aberration detection analysis of those data for identification of and timely response to multistate outbreaks. Further evaluation of the factors that contribute to NNDSS reporting timeliness is warranted.

## Background

Public health surveillance is defined as the "ongoing systematic collection, analysis, and interpretation of data essential to the planning, implementation, and evaluation of public health practice, closely integrated with the timely dissemination of these data to those who need to know"[1]. Reasons for conducting public health surveillance can include the need to assess the health status of a population, establish public health priorities, and reduce the burden of disease in a population by appropriately targeting effective disease prevention and control activities [2].

Timeliness is a key surveillance system metric and should be periodically evaluated [3,4] because it can reflect the time delay between any number of response steps in the public health surveillance process. Surveillance system timeliness depends on a number of factors and its assessment should include a consideration of how the data will be used and the nature of the condition under surveillance (e.g., for infectious diseases, this includes the communicability of the disease) [3]. If the data are to be used to implement immediate disease control and prevention activities for infectious diseases that are acute, severe, and highly transmissible, timeliness is critical. Timeliness requirements for a surveillance system might vary by different levels of public health system (e.g., local, state, or national), on the basis of the intended uses of the surveillance data at that level (Table 1). For example, timely data are needed within a state for identifying cases or clusters of disease that will prompt an immediate public health response. Timely national surveillance data aggregated from a number of jurisdictions may be used for identifying multistate outbreaks or disease clusters and enable the federal public health system to assist the states in performing and coordinating their prevention and control activities. In reportable disease surveillance systems, health care providers and diagnostic laboratories usually report information regarding persons with notifiable conditions to the local public health system. Then, reporting proceeds in a hierarchical fashion to the state and then to the national level. Health care provider and public health system actions at each successive level of the reporting hierarchy contribute to reporting timeliness delays at the national level.

**Table 1:** Potential uses of infectious disease surveillance data, by level of the public health system

Intended Uses	Used at which level(s) of the public health system?*
Identify individual cases or clusters in a jurisdiction to prompt intervention or prevention activities	Local, State (National)
Identify multi-state disease outbreaks or clusters.	State, National
Monitor trends to assess the public health impact of the condition under surveillance.	State, National (Local)
Demonstrate the need for public health intervention programs and resources, as well as allocate resources.	State, National (Local)
Monitor effectiveness of prevention, control, and intervention activities.	State, National (Local)
Formulate hypotheses for further study.	National (State)

*Public health system level in parentheses represents secondary use of the data for that purpose.

### State and national surveillance processes

**Figure 1:**
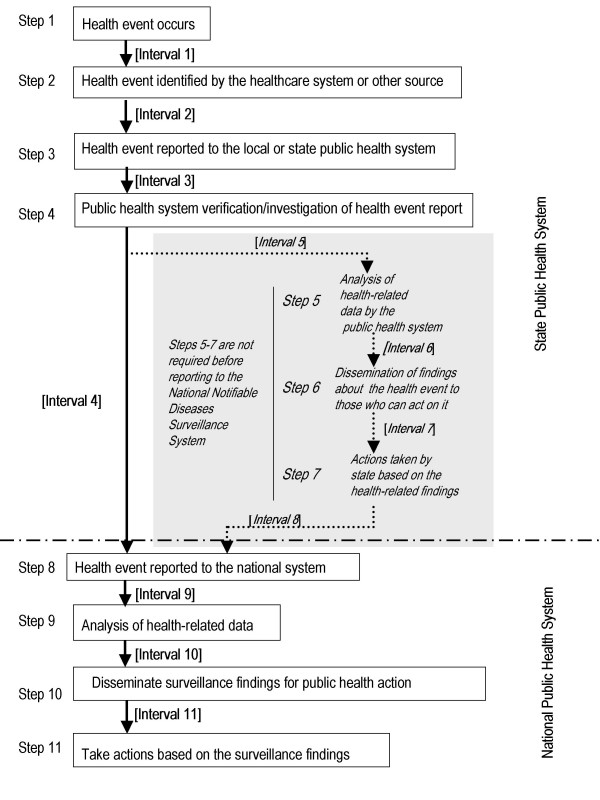
Sequence of actions needed to gather and use health-related information for public health purposes

Before data can be used for public health action, health-related data must be collected by the public health system, analyzed, and disseminated to those responsible for taking action (Figure 1). Within a state (Steps 1–7), the public health system can use surveillance data for a number of purposes, including outbreak detection and intervention planning and implementation (Table 1). The number and sequence of actions a state conducts before reporting data to the national public health system might vary by state, depending on state policies and protocols (Figure 1). For example, for nationally notifiable infectious disease reporting, CDC recommends that states report as soon as they first receive information about a suspect, probable, or confirmed case. However, some states only report confirmed cases, which usually requires laboratory confirmation, and decreases reporting timeliness at the national level.

Each week, states and the U.S. territories report case information on persons suspected of having or diagnosed with a nationally notifiable infectious disease to the Nationally Notifiable Diseases Surveillance System (NNDSS), maintained by the Centers for Disease Control and Prevention (CDC) [5]. A nationally notifiable disease is one for which "regular, frequent, and timely information regarding individual cases is considered necessary for prevention and control of the disease" [6]. At the national level, NNDSS data are used for monitoring trends, program planning, evaluation, policy development, research, and monitoring the effectiveness of prevention and control activities. Although NNDSS reporting timeliness for these long-range goals and objectives is not critical, the threat of terrorism prompted consideration of whether NNDSS could be enhanced in the future to support public health response for either naturally occurring diseases or terrorism preparedness and response efforts. Therefore, the timeliness of NNDSS data was evaluated to determine if NNDSS could support timely notification and state response to multistate outbreaks. To provide a context for the evaluation of NNDSS timeliness, published studies reporting timeliness measures for infectious disease surveillance systems in the United States were reviewed.

## Methods

### Literature review

Infectious disease surveillance evaluation studies reporting timeliness measures that were published between January 1970 and March 2003 in biomedical and public health literature were reviewed. English-language papers were identified by using the Medline database (U.S. National Library of Medicine). The search strategy used various combinations of the following key words "timeliness," "reporting delay," "time delay," "lag time," "disease surveillance," "disease outbreaks," "communicable diseases," and "infectious diseases." Reference lists of the studies identified through the Medline search and studies citing CDC's surveillance evaluation guidelines were also reviewed [3,7]

Reports were included if they evaluated a public health surveillance system operating in the United States and provided a quantitative estimate of disease-specific timeliness (e.g., interval in days). Studies without quantitative timeliness estimates or that reported a quantitative estimate for a group of infectious diseases (versus a disease-specific estimate) were excluded. In addition, studies describing the timeliness of syndromic surveillance systems were excluded.

Information abstracted for the review included the disease(s) under surveillance, the geographic area and time period studied, the purpose of the surveillance evaluation, the surveillance time interval measured, the surveillance processes or actions (steps in Figure 1) covered within the measured time interval, the timeliness measure, and the study's assessment of whether surveillance data timeliness met the surveillance goals.

### NNDSS timeliness

Information available for assessing NNDSS reporting timeliness includes the *Morbidity and Mortality Weekly Report *[*MMWR*] week number the state assigns to each case and one of the following earliest known dates associated with the incidence of this disease (earliest known date) from the following list of hierarchical date types: onset date, diagnosis date, date of laboratory result, or date of first report to the community health system. National reporting delay was calculated as the difference in days between the midpoint of the *MMWR *week and the earliest known date reported in association with the case. This time interval reflects various state-specific surveillance intervals in the surveillance process that occur between the occurrence of a health event and the reporting of that health event to NNDSS, but at a minimum it includes Intervals 1–4 (Figure 1). National median reporting timeliness was calculated overall for the years 1999–2001, for each disease in our study, by date type and state, and across all states. Median reporting delay was calculated using Proc means in SAS version 8 software for Windows (SAS Institute, Inc., Cary, North Carolina).

To assess whether analysis of NNDSS data could support the timely identification of multistate outbreaks at the national level, the percentage of NNDSS cases reports reported within one to two incubation periods for each of the diseases was determined. Incubation periods were used as a surrogate measure for period of communicability which is critical to consider when implementing effective, disease-specific prevention and control measures. For this analysis, estimated incubation periods were used for the seven nationally notifiable infectious diseases selected for this study: 7 days for cryptosporidiosis, 4 days for *Escherichia coli *O157:H7 (*E. coli*), 30 days for acute hepatitis A virus infection, 4 days for meningococcal disease, 20 days for pertussis, 1.5 days for salmonellosis, and 3 days for shigellosis [8]. These diseases were selected because they were confirmed on the basis of laboratory criteria; they have the potential to occur in epidemics; they were designated nationally notifiable five years or more before the study period began; and the magnitude of reported disease incidence supported this analysis.

Only finalized case-specific data reported from U.S. states and two autonomous reporting entities (New York City and Washington D.C., referred to as states, hereafter) that designated the reported condition as notifiable (reportable by law or regulation) and that met NNDSS publication criteria [9] were included in the analysis. Data were analyzed for *MMWR *years 1999, 2000, and 2001.

## Results

### Literature review

Eight papers were identified that met the inclusion criteria for this study (Table 2 - see Additional file: 1) [10-17]. Seven of the eight papers met the inclusion criteria resulting from the literature review; an additional paper was identified from the review of reference lists of studies identified through the Medline search and studies citing CDC's evaluation guidelines [3,7]. Three of the eight papers in this study assessed national reporting timeliness; the remaining five papers focused on local or state reporting timeliness. The studies of national reporting timeliness focused on the following diseases: acquired immunodeficiency syndrome (AIDS) [17]; *Neisseria meningitidis *and *Haemophilus influenzae *infections [16]; and shigellosis, salmonellosis, hepatitis A, and bacterial meningitis [11]. The studies of local or state reporting timeliness analyzed data for AIDS [14,15], tuberculosis [13], influenza-like illness [10], and meningococcal disease [12]. In seven of the eight papers, timeliness was calculated as the median reporting delay between the date of disease occurrence (e.g., disease onset date, diagnosis date, or laboratory result date) and the date the public health system was notified or as the proportion of cases reported to the public health system in a specific time interval. In one study [10], epidemic curves were compared for two influenza surveillance systems and timeliness was assessed as the time interval between the epidemic peaks noted in each system. In addition, two studies described the factors associated with delayed reporting [13,15].

Seven of the eight studies addressed whether the calculated timeliness measure met the needs of the surveillance process being evaluated [10,12-17]. Measured timeliness was compared with recommended reporting timeliness in two papers – a national recommendation for local tuberculosis reporting timeliness [13] and a state mandate for reporting meningococcal disease cases to local public health [12]. The adequacy of the timeliness measure for the surveillance purpose was also assessed in other ways: 1) by comparing the timeliness of the same surveillance interval in an AIDS surveillance system before and after a major revision in the AIDS surveillance case definition [17], 2) by comparing the timeliness of the same surveillance interval across an active and a passive AIDS surveillance system [14], 3) by comparing outbreak detection abilities of an existing sentinel health care provider-based surveillance system for influenza-like illness with a new school-based system monitoring illness absenteeism [10], 4) by assessing whether reporting timeliness for *Neisseria meningitidis *and *Haemophilus influenzae *was adequate to initiate a rapid public health response [16], and 5) by comparing the timeliness of reporting by whether the case-patient's initial AIDS-defining condition was included in the 1997 or 1993 AIDS surveillance case definition [15].

The reporting timeliness of AIDS and bacterial meningitis (including meningococcal disease) surveillance systems were more frequently assessed than those for other infectious diseases. The AIDS reporting timeliness studies indicate that local and national AIDS reporting timeliness meets the goals of the AIDS surveillance systems monitoring trends, targeting prevention programs, estimating needs for medical and social services, and allocating resources [14,15,17]. Timeliness of AIDS surveillance improved after the revision of the AIDS surveillance case definition in 1993 [14,15,17]. Evaluation of Tennessee's *Neisseria meningitidis *infection surveillance system for 1989–1992 indicated that the lengthy reporting interval limited the usefulness of the system for supporting rapid response for control and prevention [16]. In contrast, a 1991 evaluation of New York State's meningococcal surveillance system indicated that the majority of cases (66%) were being reported within the recommended time frame (i.e., within one day of the diagnosis to ensure chemoprophylaxis for exposed persons) and therefore, supported prevention and control efforts [12]. In addition, on the basis of nationally notifiable infectious disease data from 1987, bacterial meningitis had the shortest reporting timeliness (median 20 days) of the other infectious diseases studied [11].

The definition of reference dates used in the timeliness evaluations varied. The initial date associated with the case varied among date of disease onset, date of diagnosis, and date of positive culture result. The ending date for the timeliness studies evaluated was the date the case report was received by the public health system, whether at the local, state, or national level. This time period corresponds to the sum of Intervals 1 and 2 or Interval 2 alone for local or state timeliness studies (Figure 1). For national evaluations of timeliness, the time period assessed was the sum of Intervals 1, 2, 3, and 4 or only Intervals 2, 3, and 4 (with or without inclusion of Intervals 5, 6, 7, and 8, dependent upon state protocol).

### NNDSS timeliness

For *MMWR *years 1999–2001, a total of 9,276 cases of cryptosporidiosis, 12,332 cases of *E. coli *O157:H7 infection, 41,058 cases of hepatitis A virus acute infection, 7,090 cases of meningococcal disease, 22,735 cases of pertussis, 120,688 cases of salmonellosis, and 60,693 cases of shigellosis and were reported to NNDSS. Of those, 7,079 (76.3%) cryptosporidiosis case reports, 9,674 (78.4%) case reports of *E. coli *O157:H7 infection, 32,953 (80.3%) case reports of acute hepatitis A virus infection, 5,580 (78.7%) case reports of meningococcal disease, 19,904 (87.5%) case reports of pertussis, 84,746 (70.2%) case reports of salmonellosis, and 41,643 (68.6%) case reports of shigellosis were eligible for analysis. A total of 72,293 (26.4%) case reports were excluded for one or more of the following reasons: reported as a summary or aggregate record in which individual cases may have different event dates (20,194 cases), unknown or missing date types (20,019 cases), date type coded to *MMWR *report date (11,851 cases), and calculated reporting lag had a value of zero (indicating the event date and midpoint of the *MMWR *week matched) or had a negative value (indicating the event date was later than the mid-point of the *MMWR *week [67,557 cases]).

Timeliness of reporting varied by disease and date type (Table 3). For cases reported with a disease onset date, the median reporting delay across all reporting states varied from 12 days for meningococcal disease to 40 days for pertussis. For cases reported with a laboratory result date, median reporting delay varied from 10 days for both meningococcal disease and shigellosis to 19 days for pertussis. There was also substantial variation in state-specific median reporting delays for each disease (Table 3). For example, for meningococcal disease cases reported with a laboratory result date, state-specific median reporting delay varied from a median of 2 days in one state to 117 days in another.

**Table 3:** Timeliness of reporting of selected nationally notifiable infectious diseases, by date type, NNDSS, 1999–2001

	**Date type (Intervals from Figure 1)**			
	
Disease (incubation period*), Characteristic	Disease onset (Intervals 1,2,3,4)	Diagnosis date (Intervals #2,3,4)	Lab result date (Intervals #2,3,4)	Date of first report to the community health system (Intervals #3,4)
**Cryptosporidiosis **(7 day incubation period)				
Median time interval (days)	22	14	13	26
State-specific reporting range^a^	2–149	1–73	2–58	1–53
No. cases	4,130	956	1,825	168
No. states	44	24	41	15
% within 1, 2 incubation periods^b^	24%, 39%	37%, 50%	35%, 54%	19%, 33%
***E. Coli *O157:H7 **(4 day incubation period)				
Median time interval (days)	17	21	11	15
State-specific reporting range^a^	2–81	2–41	1–53	1–49
No. cases	6,891	473	2,206	104
No. states	48	22	39	14
% within 1, 2 incubation periods^b^	15%, 27%	13%, 25%	19%, 39%	21%, 33%
**Hepatitis A, acute **(30 day incubation period)				
Median time interval (days)	23	18	12	12
State-specific reporting range^a^	2–54	2–80	2–29,231^+^	1–126
No. cases	21,570	4,394	6,695	294
No. states	49	36	39	14
% within 1, 2 incubation periods^b^	62%, 84%	67%, 83%	82%, 94%	79%, 91%
**Meningococcal disease **(4 day incubation period)				
Median time interval (days)	12	13	10	10
State-specific reporting range^a^	2–56	1–54	2–117	4–62
No. cases	3,804	450	1,255	71
No. states	50	30	39	7
% within 1, 2 incubation periods^b^	23%, 39%	26%, 40%	25%, 44%	31%, 42%
**Pertussis **(20 day incubation period)				
Median time interval (days)	40	31	19	23
State-specific reporting range^a^	2–124	1–106	2–190	2–48
No. cases	18,750	289	758	107
No. states	50	26	34	15
% within 1, 2 incubation periods^b^	24%, 50%	34%, 60%	53%, 78%	45%, 68%
**Salmonellosis **(1.5 day incubation period)				
Median time interval (days)	17	7	12	16
State-specific reporting range^a^	2–44	1–54	2–61	1–27
No. cases	49,659	5,558	28,172	1,357
No. states	47	35	42	28
% within 1, 2 incubation periods^b^	4%, 13%	17%, 43%	6%, 17%	7%, 19%
**Shigellosis **(3 day incubation period)				
Median time interval (days)	15	10	10	9
State-specific reporting range^a^	2–43	1–51	2–34	1–26
No. cases	26,635	2,850	11,603	555
No. states	46	28	41	17
% within 1, 2 incubation periods^b^	15%, 22%	33%, 39%	22%, 35%	29%, 41%

*Source: Control of Communicable Diseases Manual 17^th ^Edition [8]. ^+^The maximum state-specific median reporting delay for this disease and date type is from a state that reported 19 cases having event years 1919 or 1920. Excluding these cases as data entry errors, the maximum state-specific median reporting delay is 78 days. ^a^State-specific median reporting range (minimum, maximum) in days ^b^% of cases reported within 1 and 2 incubation periods, respectively

For the same date type, NNDSS diseases with longest incubation periods tended to have a higher percentage of cases reported within one or two incubation periods than NNDSS diseases with shorter incubation periods (Table 3). For example, for acute hepatitis A virus infection, which had the longest incubation period of all the study diseases, more than 60% of cases were reported within one incubation period, for each date type reported. For all other diseases except pertussis, less than 40% of cases were reported within one incubation period for each reported date type. For pertussis, the percentage of cases reported within one incubation period varied from 24% for reports with disease onset date to 53% for case reports with laboratory result dates. In addition, state-specific percentage of cases reported within one or two incubation periods varied for a given disease and date type (data not shown).

### Comparison of NNDSS timeliness and literature review results

The 1999–2001 NNDSS meningococcal disease median reporting interval between date of disease onset and date of report to CDC in this study was 8 days shorter than a previous study reported [11] using 1987 notifiable disease data for bacterial meningitis (median 20 days); and, the meningococcal disease median reporting delay was 9 days shorter in this study than in a previous study [16] using Tennessee's data for the years 1989–1992 for *Neisseria meningitidis *infection (median 21 days). In addition, the median reporting delay between disease onset and the date of report to CDC was shorter in this study than in a previous study (which used 1987 notifiable disease data) by 10 days for hepatitis A, 5 days for salmonellosis, and 8 days for shigellosis [11].

## Discussion

Few published studies evaluating surveillance systems presented timeliness measures. When timeliness was evaluated, standard methods were not used. Information collected by public health surveillance systems should support the quantitative assessment of timeliness by various steps in the pubic health surveillance process. Public health programs should periodically assess timeliness of specific steps in the surveillance system process to ensure that the objectives of the surveillance system are being met. A more structured approach to describing timeliness studies should be considered.

Published papers describing local or state surveillance system reporting timeliness generally do not explicitly describe the surveillance system processes contributing to the timeliness measure, such as processing and analyzing the data or implementing a public health action before data are reported from a state to CDC. To facilitate future comparisons of reporting timeliness across jurisdictions, studies should include an explicit description of the public health surveillance reporting process and the surveillance process interval being measured. Additionally, surveillance information systems must support the collection of appropriate reference dates to allow the assessment of the timeliness of specific surveillance processes.

A more structured approach to describing timeliness studies could include a description of the following characteristics: 1) the level of the public health system being assessed (e.g., local, state, or national), 2) the purpose of the surveillance evaluation, 3) goals of the surveillance system, 4) the surveillance interval being measured and a description of the reference dates that define the upper and lower boundaries of the surveillance interval, 5) the surveillance steps (processes or activities) that contribute to the surveillance interval being measured, 6) whether the measured timeliness met the needs of the surveillance step being evaluated, and 7) whether the timeliness met the goals of the surveillance system. No single timeliness measure will achieve the purpose of all evaluations or meet all the goals of the surveillance system. In addition, if the goal of the surveillance evaluation is to identify ways to improve timeliness, the analysis should identify factors associated with delayed reporting, such as the role of specific case ascertainment sources.

The 1999–2001 national notifiable diseases data were timely enough to support the following surveillance objectives: monitoring trends over time, informing allocation of public health resources, monitoring the effectiveness of disease control, identifying high risk populations, and testing hypotheses. If NNDSS data are to be used to support timely identification of and response to multistate outbreaks at the national level, the timeliness of reporting needs to be enhanced for all diseases, but especially for diseases with the shortest incubation periods (e.g., cryptosporidiosis, *E. coli *O157:H7, meningococcal disease, salmonellosis, and shigellosis). Until reporting timeliness is enhanced, the application of aberration detection analytic methods to NNDSS data to aid in the identification of changes in disease reporting that may indicate a multistate outbreak in time to alert states for the purposes of disease control and prevention may be of limited use. Future work to improve reporting timeliness will need to address the substantial variation across states. As states enhance their reporting mechanisms with the use of automated electronic laboratory reporting systems [18], there may be less variation in state-specific reporting timeliness, but this should be assessed.

NNDSS timeliness improved compared to timeliness of notifiable infectious diseases measured in previous reports [11,16]. However, the methods or variables used in these analyses were different. A few factors may have contributed to improvements in timeliness seen in this study. Since 1992, states have been routinely transmitting electronic case-specific records intended to improve reporting procedures and protocols. In addition, the use of automated electronic laboratory reporting to enhance infectious disease case reporting may have contributed to increased timeliness.

Our study findings are subject to several limitations. The variables available for assessing NNDSS reporting timeliness are based on the *MMWR *week numbers that are assigned by states and the earliest known date reported in association with the case. While these variables might provide an estimate of national reporting timeliness, NNDSS data do not include a fixed date defining when a case report was initially transmitted to CDC or received at CDC, which would provide a more precise measure of national reporting timeliness. NNDSS data management protocols should be modified to permit direct calculation of national reporting timeliness. If the ability to support outbreak detection at the national level using NNDSS data is generally viewed as an important and sustainable enhancement for the NNDSS, states and CDC programs should facilitate reporting that more closely approximates real-time and define reporting protocols and data requirements to ensure that reporting timeliness can be improved and accurately monitored. The current NNDSS practice of weekly reporting and data processing limits reporting timeliness to CDC. Lastly, 72,293 (26.4%) cases were excluded from our analysis because the information contained in the database would not permit calculation of timeliness and this exclusion may have resulted in our study results either falsely overestimating or underestimating the magnitude of NNDSS reporting lags.

The reporting timeliness variations across states may result from different reporting protocols in the states (e.g., centralized versus distributed reporting within the state's public health system) or from variations in how states assign *MMWR *week numbers. Other factors that might have contributed to reporting delay in our study included: the patient's recognition of symptoms; the patient's acquisition of medical care; the use of confirmatory laboratory testing; reporting by the health care provider or the laboratory to the local, county, or state public health authority; the volume of cases identified in the state; case follow-up investigations to verify the case report or to collect additional case information; periods of decreased surveillance system activity due to variable staffing levels; computer system down-time for maintenance, upgrades, or new application development; and data processing routines, such as data validation or error checking. Following a structured approach to evaluation of timeliness by specifying the surveillance objectives and the process(es) being measured may allow better definition of the factors that contribute to reporting delay. It was beyond the scope of this study to assess how these factors contribute to NNDSS reporting timeliness.

In addition to reporting timeliness, other surveillance system attributes are important to assess (e.g., completeness of reporting). Completeness of notifiable infectious diseases reporting in the United States varies from 9% to 99% [7]. Six of the eight papers reviewed for this study assessed completeness of reporting [12-17]. One paper [14] noted that although the timeliness of the AIDS passive and active surveillance systems were comparable, the completeness of the active AIDS reporting system far exceeded the reporting completeness for the passive system. This highlights the importance of evaluating completeness and timeliness and other surveillance system attributes concurrently, before contemplating any changes to a surveillance system based on the assessment of a single attribute.

To improve public health surveillance infrastructure and performance in the United States, CDC and local and state health agencies are integrating a number of public health surveillance systems monitoring infectious diseases in the United States, including the NNDSS, into the National Electronic Disease Surveillance System (NEDSS) [19,20]. NEDSS outlines a standards-based approach to disease surveillance and intends to connect public health surveillance to the clinical information systems infrastructure. As a result, NEDSS promises to improve the accuracy, completeness, and timeliness of disease reporting to state and local health departments and CDC.

## Conclusions

To facilitate comparisons of surveillance system timeliness studies across jurisdictions or health conditions, a more standardized approach to describing timeliness studies is warranted. Public health surveillance systems should ensure that timeliness can be measured for specific surveillance system processes and in the context of the goals of surveillance. In addition, when timeliness is being measured, it is important to be explicit about how it is being measured. Our analysis of NNDSS reporting timeliness suggests that current acute hepatitis A infection reporting timeliness may be sufficient to support a timely public health response in the event of a multistate outbreak. However, for the other conditions evaluated, the long reporting lag and the variability across states limits the usefulness of NNDSS data and aberration detection analysis of those data for identification of and response to multistate outbreaks. The NNDSS timeliness data presented in this paper represents a baseline against which timeliness can be measured in the future. Further study is needed to identify the major sources of reporting delay and to assess how NNDSS reporting timeliness may be improved for the timely detection of cases and disease clusters.

## Competing interests

None declared.

## Author's contributions

Both authors contributed equally to project conception and write-up of the manuscript. RAJ was responsible for data analysis. Both authors read and approved the final manuscript.

## Pre-publication history

The pre-publication history for this paper can be accessed here:



## Supplementary Material

Additional File 1 Published reports quantitatively measuring timeliness of reporting infectious disease surveillance data. Table summarizes the findings of the review of published literature about quantitative measurements of infectious disease surveillance system timeliness Click here for file

## References

[B1] Thacker SB, Berkelman RL (1988). Public health surveillance in the United States. Epidemiol Rev.

[B2] Thacker SB, Teutsch SM, Churchill RE (2000). Historical development. In Principles and Practice of Public Health Surveillance.

[B3] Centers for Disease Control and Prevention (2001). Updated guidelines for evaluating public health surveillance systems: Recommendations from the Guidelines Working Group. MMWR.

[B4] Centers for Disease Control (1988). Guidelines for evaluating surveillance systems. MMWR.

[B5] Koo D, Wetterhall SF (1996). History and current status of the National Notifiable Diseases Surveillance System. Journal of Public Health Manag Pract.

[B6] Centers for Disease Control and Prevention (2001). Summary of Notifiable Diseases – United States, 2001. MMWR.

[B7] Doyle TJ, Glynn MK, Groseclose SL (2002). Completeness of notifiable infectious disease reporting in the United States: An analytical literature review. Am J Epidemiol.

[B8] James Chin (2000). Control of Communicable Diseases Manual.

[B9] Centers for Disease Control and Prevention (2004). Nationally notifiable infectious diseases event (disease or condition) code list with print criteria. http://www.cdc.gov/epo/dphsi/phs/infdis.htm.

[B10] Lenaway DD, Ambler A (1995). Evaluation of a school-based influenza surveillance system. Public Health Rep.

[B11] Birkhead G, Chorba TL, Root S, Klaucke DN, Gibbs NJ (1991). Timeliness of national reporting of communicable diseases: The experience of the National Electronic Telecommunications System for Surveillance. Am J Public Health.

[B12] Ackman DM, Birkhead G, Flynn M (1996). Assessment of surveillance for meningococcal disease in New York State, 1991. Am J Epidemiol.

[B13] Curtis AB, McCray E, McKenna M, Onorato IM (2001). Completeness and timeliness of tuberculosis case reporting: A multistate study. Am J Prev Med.

[B14] Hsu L, Schwarcz S, Katz M (2000). Comparison of simultaneous active and passive AIDS case reporting in San Francisco [Letters To The Editor]. J Acquir Immune Defic Syndr.

[B15] Schwarcz SK, Hsu LC, Parisi MK, Katz MH (1999). The impact of the 1993 AIDS case definition on the completeness and timeliness of AIDS surveillance. AIDS.

[B16] Standaert SM, Lefkowitz LB, Horan JM, Hutcheson RH, Shaffner W (1995). The reporting of communicable diseases: A controlled study of *Neisseria meningitidis *and *Haemophilus influenzae *infections. Clin Infect Dis.

[B17] Klevens RM, Fleming PL, Li J, Gaines CG, Gallagher K, Schwarcz S, Karon JM, Ward JW (2001). The completeness, validity, and timeliness of AIDS surveillance data. Annals of Epidemiol.

[B18] Effler P, Ching-Lee M, Bogard A, Ieong MC, Nekomoto T, Jernigan D (1999). Statewide system of electronic notifiable disease reporting from clinical laboratories: Comparing automated reporting with conventional methods [erratum appears in JAMA 2000 Jun 14;283(22):2937]. JAMA.

[B19] Centers for Disease Control and Prevention (2003). National Electronic Disease Surveillance System (NEDSS). http://www.cdc.gov/nedss/.

[B20] National Electronic Disease Surveillance Systems Working Group (2001). National Electronic Disease Surveillance System (NEDSS): A standards-based approach to connecting public health and clinical medicine. Journal of Public Health Manag Pract.

