# Supplementary material for: Evaluation of reporting timeliness of public health surveillance systems for infectious diseases
Source: BMC Public Health. 2004 Jul 26;4:29. doi: 10.1186/1471-2458-4-29 (PMC509250; doi:10.1186/1471-2458-4-29)
Supplement: Additional File 1 — Published reports quantitatively measuring timeliness of reporting infectious disease surveillance data. Table summarizes the findings of the review of published literature about quantitative measurements of infectious disease surveillance system timeliness [file 1471-2458-4-29-S1.doc]

Table 2. Published reports quantitatively measuring timeliness of reporting infectious disease surveillance data

| **First Author** | **Location** | **Time period** | **Purpose of the surveillance evaluation** | **Surveillance interval measured** | **Public health action intervals measured (see Figure 1)** | **Disease (s)** | **Timeliness metric and measure** | **Did study address whether timeliness met the needs of the step evaluated, and if so, the result.** | **Did timeliness meet surveillance goal?** |
| --- | --- | --- | --- | --- | --- | --- | --- | --- | --- |
| Hsu [14] | San Francisco, California | 1995-1997 | Compare reporting timeliness (and completeness) between active and passive surveillance systems | Diagnosis date to date reported to local public health | Interval 2 | Acquired Immuno-deficiency Syndrome (AIDS) | Median reporting delay between date of AIDS diagnosis and date of case report for active surveillance system was 2 months (1,019 cases) and for the passive surveillance system was 1 month (56 cases). The proportion of cases reported to local public health within 6 months of the AIDS diagnosis was 82% in the active surveillance system and 84% by the passive system. | Yes, reporting timeliness by the passive surveillance system was comparable to reporting timeliness by the active system (however, reporting completeness in the passive system was poor compared to the active system). | Yes, timeliness was adequate for planning prevention activities and for allocating health service funding. |
| Klevens [17] | San Francisco, California; Massachusetts; Louisiana | 1991, 1995 | Assess whether reporting timeliness (completeness and data validity) changed after the 1993 AIDS case definition revision | Diagnosis date to date reported to national public health system | Intervals 2-4; or, Intervals 2 -3 and 5-8 | AIDS | Median reporting delay for interval between month and year of the AIDS diagnosis and month and year of report to CDC (statistical significance for the difference in delay from 1991 to 1995): San Francisco: 14 months in 1991 and 3 months in 1995 (p=0.0001). Massachusetts: 7 months in 1991 and 6 months in 1995 (p=0.367). Louisiana: 2 months in 1991 and 3 months in 1995 (p=0.003). Overall: 6 months in 1991 and 4 months in 1995 (p=0.0001) | Yes, overall reporting timeliness improved (and reporting completeness and validity remained high) after the 1993 AIDS case definition revision. | Yes, timeliness improved overall (and reporting complete-ness and accuracy remained high), after the 1993 case definition revision, which is important for character-  izing the AIDS epidemic and applying the results to disease prevention. |
| Schwarcz [15] | San Francisco, California | 1994 | Assess the impact of a change in case definition on reporting timeliness (and completeness) | Diagnosis date to date reported to local public health | Interval 2 | AIDS | Median time interval between date of diagnosis and date of the case report: 1 month (433 cases). Median reporting delay was calculated for characteristics of the cases, diagnosing facilities, and other factors. Independent predictors of reporting delay were also calculated. | Yes, cases meeting the 1993 AIDS case definition were reported 2 months earlier than cases meeting the 1987 AIDS case definition. Case reporting from private physicians was less timely than case reporting from clinics and hospitals. | Yes |
| Lenaway [10] | Boulder, Colorado | 1988-1993 | Compare outbreak detection performance between two surveillance systems | Diagnosis report to county public health system (passive sentinel system); school-based illness absenteeism report to county public health system | School-based system, Intervals 1-3; Passive sentinel system based on diagnosis, Interval 2 | Influenza-like illness (sentinel system); school-based excessive illness absenteeism | Comparison of epidemic curves between two surveillance systems—a school-based surveillance system which recorded weekly illness absenteeism and reported summary counts to public health when the weekly average absenteeism rate exceeds 7.5 percent of the school census and a pre-existing sentinel surveillance system for influenza-like illness. In 2 of the 5 flu seasons evaluated, school-based system peaked 1 week prior to physician-based system. | Yes, the school-based system allowed the local health department to track influenza-like illness activity and provide timely and important information about outbreaks to the community, schools, health care providers, and public health specialists. | Yes |
| Ackman [12] | New York State (excluding New York City) | 1991 | Assess timeliness (and completeness) of case reporting for effective prevention and control | Diagnosis date to date reported to local public health | Interval 2 | Meningococcal disease | Reporting delay between date of diagnosis and date of notification for 80 cases was calculated and the percentage of cases reported within a specific time period was reported: 39% reported the day of diagnosis; 66% reported within 1 day of diagnosis. | Yes, the majority of cases are reported within 24 hours of diagnosis, as required by New York State in order to ensure chemopro-phylaxis is given to all exposed persons. However, there was a delay in reporting some cases. | Yes |
| Standaert [16] | Tennessee | 1989-1992 | Compare case-finding capabilities of a passive surveillance system with those of an active laboratory-based system. Assess whether passive system could be used to direct a rapid response. | Laboratory report date to date reported to national public health system | Intervals 2-4; or, Intervals 2-3 and 5-8 | *Neisseria meningitidis* infection, *Haemophilus influenzae* infection | Median (minimum, maximum) reporting interval between date of positive culture result (disease onset) and date the state reported data to CDC: *N. meningitidis*: 21 (5, 124 ) days, 41 cases; *H. influenzae*: 25 (11, 157) days, 94 cases; For meningitis cases diagnosed and reported by a physician, median reporting delay was 61 (31, 124) days. | Yes, the lengthy reporting timeliness in the passive surveillance system doesn't permit it to function to direct a rapid response. | No, alternative mechanisms for rapid case detection, such as laboratory-based reporting using computer telecom-munications, are needed to help direct immediate control and prevention efforts. |
| Birkhead [11] | United States | 1987 | Assess reporting delay of selected communicable diseases to the federal public health system | Onset date to date reported to federal public health | Intervals 1-4; or, Intervals 1-3 and 5-8 | Shigellosis, Salmonellosis, Hepatitis A, Bacterial meningitis | Median reporting delay between disease onset and date of report to CDC, in days (number of cases, number of states reporting): shigellosis: 23 days (7,167 cases, 23 states) salmonellosis: 22 days (1,7624 cases, 22 states) hepatitis A: 33 days (9,592 cases, 25 states) bacterial meningitis: 20 days (1,241 cases, 28 states). Median reporting delays for salmonellosis and shigellosis varied by state from 4 days in one state to over 8 weeks for another state. | No | Not addressed by the study |
| Curtis [13] | California; Florida; Massachuesetts; New Jersey; New York State, excluding New York City; Washington | 1993-1994 | Assess whether data are timely (and complete) enough for effective disease control | Diagnosis date to date reported to local or state public health | Interval 2 | Tuberculosis | Median reporting delay varied by site and ranged from a median of 7 days for 352 cases in New Jersey to a median of 38 days for 684 cases in Florida.  Percentage of cases reported within 3 days varied by site, but was 20% (520 of 2,591 cases) across all sites. Percentage of cases reported within 7 days was 29% (741 of 2,591 cases) and also varied by site.  Factors associated with delayed reporting also presented. | Yes, reporting timeliness was poor among the reported cases, as determined by comparing the study results to the CDC recommendation that TB cases be reported to local health departments within 2 working days of the TB diagnosis. | No, delays in reporting timeliness hinder timely assessment and treatment of contacts and timely identification of secondary cases. |
